# Advancements and Challenges in Immune Protection Strategies for Islet Transplantation

**DOI:** 10.1111/1753-0407.70048

**Published:** 2025-01-20

**Authors:** Xue Wang, Ziyuan Zeng, Dayan Li, Kai Wang, Wei Zhang, Yang Yu, Xi Wang

**Affiliations:** ^1^ State Key Laboratory of Female Fertility Promotion, Department of Obstetrics and Gynecology, Clinical Stem Cell Research Center Peking University Third Hospital Beijing China; ^2^ Department of Physiology and Pathophysiology, School of Basic Medical Sciences, State Key Laboratory of Vascular Homeostasis and Remodeling Clinical Stem Cell Research Center, Peking University Third Hospital, Peking University Beijing China; ^3^ Beijing Advanced Center of Cellular Homeostasis and Aging‐Related Diseases Peking University Beijing China; ^4^ TianXinFu (Beijing) Medical Appliance co. Ltd. Beijing China; ^5^ Institute of Advanced Clinical Medicine, Peking University Beijing China

**Keywords:** encapsulation, immune rejection, immunomodulatory strategies, pancreatic islet transplantation, type 1 diabetes (T1D)

## Abstract

Pancreatic islet transplantation is a crucial treatment for managing type 1 diabetes (T1D) in clinical settings. However, the limited availability of human cadaveric islet donors and the need for ongoing administration of immunosuppressive agents post‐transplantation hinder the widespread use of this treatment. Stem cell‐derived islet organoids have emerged as an effective alternative to primary human islets. Nevertheless, implementing this cell replacement therapy still requires chronic immune suppression, which may result in life‐long side effects. To address these challenges, innovations such as encapsulation devices, universal stem cells, and immunomodulatory strategies are being developed to mitigate immune rejection and prolong the function of the transplant. This review outlines the contemporary challenges in pancreatic β cell therapy, particularly immune rejection, and recent progress in immune‐isolation devices, hypoimmunogenic stem cells, and immune regulation of transplants. A comprehensive evaluation of the advantages and limitations of these approaches will contribute to improved future clinical investigations. With these promising advancements, the application of pancreatic β cell therapy holds the potential to effectively treat T1D and benefit a larger population of T1D patients.

## Graphical abstract

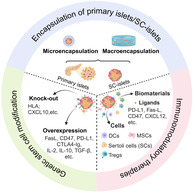

**Summary**

- Human islet transplantation improves glycemic control in type 1 diabetes (T1D), but long‐term immunosuppression and immune rejection pose significant challenges.
- Key strategies to address these issues include encapsulation technologies, gene editing of stem cells, and immunomodulation.
- These advancements promise to enhance the effectiveness and accessibility of β cell therapies for T1D patients.

## 1 Introduction

Diabetes mellitus (DM) is a chronic disease that causes a substantial socioeconomic burden globally. According to the International Diabetes Federation (IDF), an estimated 537 million adults aged 20–79 were diagnosed with diabetes in 2021 [1]. The number is projected to increase to 783.2 million by 2045, marking a 46% rise over 2021 figures [2]. Type 1 diabetes (T1D) accounts for approximately 5%–10% of all diabetes cases; however, T1D remains a critical concern due to its unique pathophysiology, early onset, and the significant challenges it presents in terms of management and treatment [3]. T1D is primarily caused by autoimmune destruction of β cells within the pancreatic islets, leading to irreversible β cell loss and insulin deficiency [4]. Current management requires daily administration of insulin injections, which cannot cure the disease and pose challenges in maintaining glycemia [5].

To enhance therapeutic outcomes and improve patients' quality of life, pancreatic islet transplantation has emerged as a promising approach with the establishment of the Edmonton Protocol in 2000 [6]. It is a minimally invasive procedure with negligible complications compared to pancreas transplantation [7, 8]. It renders T1D patients insulin‐independent for a few years, along with immunosuppressive agents. Clinical evidence also shows that patients who undergo islet transplantation therapy can maintain better blood glucose levels within the normal range with minimal disturbance compared to conventional insulin injections. Food and Drug Administration (FDA) approved the application of pancreatic islet transplantation in clinics in 2023 [9], showing the efficacy of this therapy. However, this therapy faces several challenges: (1) Limited availability of primary human pancreatic islets for transplantation [10]; (2) loss of islet cells post‐transplantation due to innate inflammatory responses [11]; (3) side effects of immunosuppressants.

Given the increasing global burden of diabetes, it is essential to explore innovative strategies to address the above‐mentioned issues and enhance the engraftment of transplanted islets. Concurrently, with the advancement in the field of stem cells, it is now possible to differentiate unlimited stem cell‐derived islets (SC‐islets) in vitro and confirm their functionality. Significant progress has been achieved in developing effective strategies to improve the success of pancreatic islet transplantation or SC‐islets therapy, especially in avoiding the use of immunosuppressive drugs.

Although several reviews published before have overviewed immune rejection strategies in islet transplantation [12, 13, 14, 15], this review offers a more comprehensive analysis by integrating the current status of both primary and SC‐islets transplantation. This review article aims to elucidate the current landscape and primary challenges in protecting pancreatic islets or SC‐islets from immune rejection. Additionally, we summarized promising strategies, including biomaterial engineering, stem cell‐based engineering, and immunomodulatory engineering aimed at preventing the need for chronic immunosuppression, while also incorporating the latest clinical trial data.

## 2 Islet Transplantation

Islet transplantation is an effective treatment for diabetes. However, the shortage of donors and the challenges of immune rejection have limited its widespread adoption. Therefore, identifying alternative sources for pancreatic islet transplantation is paramount.

### 2.1 Current Status of Primary Islet Transplantation

Islet transplantation was initially tested in rat [16] and dog [17] models before its first validation in humans in 1974 [18]. However, achieving sustained independence from exogenous insulin proved to be difficult. A breakthrough was achieved in 2000 when the Edmonton Protocol was developed, and temporary insulin independence in T1D patients was observed [6]. It describes isolating human primary islets from the pancreas using a mixture of enzymes, injecting human islets isolated from 2 to 3 donors into the hepatic portal vein, and administering immune suppressants. Islet transplantation has since become a standard treatment for T1D in Canada [19], Australia, and the United States [20]. A significant milestone was achieved on June 28, 2023, with the FDA's approval of Lantidra (donislecel). This is the first allogeneic human pancreatic islet cell therapy for adults with T1D [9]. This approval marks a pivotal advancement of cell therapy to treat diabetes.

Under current clinical preparation techniques, only 30%–50% of pancreatic islets are successfully isolated, highlighting the need to improve islet isolation efficiency. Key factors such as donor selection, the islet isolation process, and pre‐transplant culture conditions play critical roles in determining transplantation outcomes. Therefore, successful islet isolation methods are critical for increasing both the yield and quality of islets while reducing the cost. The “China Clinical Islet Isolation Technical Operation Guidelines” (2023 version) provide standard procedures designed to optimize islet isolation, processing, and preservation techniques during clinical islet transplantation. Specifically, the enzyme solution used in clinical islet isolation comprises a combination of collagenase and neutral protease. Islets are separated from acinar tissue and purified through density gradient centrifugation. Following isolation, the islets can be cultured for a limited period prior to transplantation; however, the in vitro culture is generally recommended for no more than 72 h [21]. Despite significant advancements in human islet isolation and purification techniques, islet damage and functional decline remain inevitable during the isolation, purification, and culture. These factors are critical in influencing the success of clinical transplantation. Ongoing research efforts shall focus on further refining donor selection, optimizing the islet preparation process, and improving pre‐transplantation culture conditions.

In a recent single‐center cohort study with a 20‐year follow‐up, Shapiro and colleagues reported insulin independence rates of 20% at 10 years and 8% at 20 years post‐transplantation [19]. These findings indicated that recipients with islet transplant can achieve long‐term insulin independence. Data from the Collaborative Islet Transplant Registry (CITR) indicated that, compared to the levels pre‐transplantation, insulin dosage in recipients with islet transplant decreased by an average of 75%, 68%, and 64% at 1, 3, and 5‐year post‐transplantation, respectively [20]. These results suggest that islet transplantation can significantly reduce insulin requirements and, in some cases, even achieve long‐term insulin independence. Successful islet transplantation improves blood glucose control and quality of life in diabetic patients, but requires long‐term immunosuppressive therapy, which can cause side effects, and may lead to psychological stress in case of transplant failure or poor function. The outcome depends on factors such as transplantation technique, immunosuppressive management, and condition of the patient's health.

Pancreatic islet transplantation is a validated therapy approach for treating T1D, which involves progression: (1) Inhibition of the instant blood‐mediated inflammatory reaction (IBMIR), for which routine short‐term anticoagulant therapy is required [22, 23]. Recent studies, such as that by Turan A et al., have proposed co‐engineered islets incorporating thrombomodulin chimeric with streptavidin (SA‐TM) and SA‐CD47 molecules to enhance engraftment by modulating procoagulatory and inflammatory responses, thus inhibiting IBMIR [24]. (2) Local inflammation characterized by elevated cytokine production [25]. Paez Mayorga et al. introduced an innovative device called Neovascularized Implantable Cell Homing and Encapsulation (NICHE), which mitigates islet rejection by locally inducing immunosuppression [26]. (3) Prevention of recurrent autoimmune responses triggered by autoreactive immune cells is also essential [27]. In this regard, Jong‐Min Kim et al. developed an immunosuppressive regimen that promotes the long‐term survival of allo‐islets in rhesus monkeys following the rejection of prior porcine grafts [28]. (4) Successful immune tolerance refers to the ability of the immune system to recognize non‐self cells without initiating an immune rejection response. In this context, Hu et al. reported that genetically engineered, low‐immunogenic islet grafts can be successfully transplanted into non‐human primates with fully functional immune systems [29]. Despite these advancements, the shortage of pancreatic islet donors is a significant limitation that remains. Furthermore, pancreatic islet dysfunction and apoptosis, often triggered by immune rejection, continue to impact the long‐term survival of the grafts, leading to poor primary graft function [30]. Patients who received islet transplantation continue to require immunosuppressive drugs, posing three main challenges: (1) Inapplicability to all diabetes patients; (2) Long‐term use complications, including insufficient maintenance of transplanted islet viability and severe side effects, such as drug‐induced kidney injury and cardiovascular dysfunction [31, 32]; (3) Persistent risk of immunosuppressive drugs causing damage to the transplanted grafts. Consequently, there is an urgent need for innovative approaches to address primary islet immune rejection during transplantation.

### 2.2 Current Status of Cell/SC‐Islets Transplantation

Islet transplantation encounters significant challenges, including limited donor availability, immune rejection, and the need for lifelong immunosuppression [33]. Porcine islets can provide plenty of cell sources but come with an increased risk of hyperacute rejection and infections. Researchers have successfully avoided the need for immunosuppression by using donor organs from pigs with gene editing, though the effects lasted only for a few hours (or days) [34, 35]. Nizar I Mourad et al. obtained transgenic pigs expressing the glucagon like peptide 1‐type 3 muscarinic receptor cassette under the porcine insulin promoter (InsGLP‐1M3R). And InsGLP‐1M3R neonatal islets were found to be more effective in treating diabetes. Unlike previous studies that required large numbers of islets (4000–12 000 IEQ) to reverse diabetes, only 2500 IEQ were sufficient to reverse hyperglycemia over 9 months without exogenous insulin [36]. Similarly, there is a potential for reduced immune rejection with islets from gene‐edited pigs, although further investigation is needed to validate feasibility. Scientists are actively exploring solutions through genetic engineering to enhance safety and mitigate the immune rejection of porcine islets.

With advancements in stem cell technology, unlimited stem cell‐derived islets can be differentiated in vitro and proved functional in vivo in different preclinical animal models. Thus, SC‐islets emerged as a promising alternative to human primary islets. In 2017, ViaCyte conducted phase 1/2 clinical trial (VC‐02, NCT03163511) utilizing the PEC‐Encap system, which encapsulated pluripotent stem cell‐derived pancreatic endoderm cells (PECs) [37, 38, 39]. However, since the encapsulated cells are pancreatic progenitor cells rather than fully matured islet β‐cells, which may adversely affect the efficacy of the treatment. In 2021, a pharmaceutical based in Boston initiated the phase 1/2 clinical trial with fully differentiated SC‐islets (VX‐880, NCT04786262). Similar to islet transplantation, SC‐islets were administrated into the portal vein of T1D patients, alongside immunosuppressive therapy. This trial proved successful as the first T1D patient became insulin‐independent and was functional cured following SC‐islet cell therapy. Further positive data related to this trial has been released in 2023, showing 7 of 10 patients can avoid exogenous insulin completely [40, 41]. On November 4th, 2024, Vertex Pharmaceuticals made a significant announcement regarding VX‐880, revealing that the phase 1/2 trials are progressing to a phase 3 pivotal trial [42]. This is the first SC‐islets therapy targeting patients with T1D to enter phase 3 clinical trials (NCT04786262). VX‐880 employs allogeneic stem cells, which allows for pre‐preparation, and offers the potential to be engineered as universal products. Despite this advancement, VX‐880 faces several challenges, including the high cost of stem cell therapy and the necessity for long‐term immunosuppressive treatment to ensure the survival of transplanted cells. These factors may impose both financial and health burdens on patients. Additionally, the efficacy and safety of VX‐880 still require further validation through additional clinical trials. To address the adverse effects of immunosuppressive agents, Vertex has developed another therapy for treating T1D, VX‐264, which is also SC‐islets therapy but incorporates with encapsulation devices, offering an ideal therapeutic approach within a protective and retrievable device. This clinical trial is currently in phases 1/2 (NCT05791201) [43]. In addition, Shen et al. from Tianjin First Central Hospital and Deng et al. from Peking University successfully performed the transplantation of chemically induced pluripotent stem‐cell‐derived islets (CiPSC‐islets) (ChiCTR2300072200) in China. The fasting C‐peptide of T1D patients showed a significant increase (< 0.02 ng/mL vs. 0.31 ng/mL) after transplantation, and the daily insulin requirement reduced to half of that before transplantation, reaching a stable therapeutic effect within a few months. Wang and colleagues made a significant breakthrough by reporting the first case of CiPSC‐islets into T1D patient as a clinical treatment for T1D and published in Cell recently. The patient achieved insulin independence and maintained glycemic control by day 75 post‐transplantation. By day 180, the patient's HbA1c was reduced to 4.6%, and was sustained at 4.8% after one year. This marks the first report on the clinical use of CiPSC‐derived islets in the treatment of T1D [44]. On December 6th, 2024, the clinical trial application for the developed RGB‐5088 islet cell injection solution has been approved by the National Medical Products Administration (NMPA) of China (CXSL2400698) [45]. In 2024, the cell therapy utilizing autologous SC‐islets derived from endoderm stem cells (E‐islets) was performed on a patient with Type 2 diabetes (T2D) and impaired islet function in China [46]. Recently, Sernova Corporation has successfully tested Cell Pouch technology. This innovative therapy involves implanting a cell pouch loaded SC‐β cells into T1D patients, allowing them to secrete insulin and regulate blood sugar levels. It completely eliminates the daily pain of insulin injections [47, 48]. These clinical trials highlight the broad applications of this therapy beyond diabetes. However, the transplanted islets still face significant immune rejection reactions under auto‐immunogenic or allo‐immunogenic settings. Thus, addressing the immune‐related rejection in islet or SC‐islet transplantation in vivo is urgent.

## 3 Promising Strategies

**FIGURE 1:**
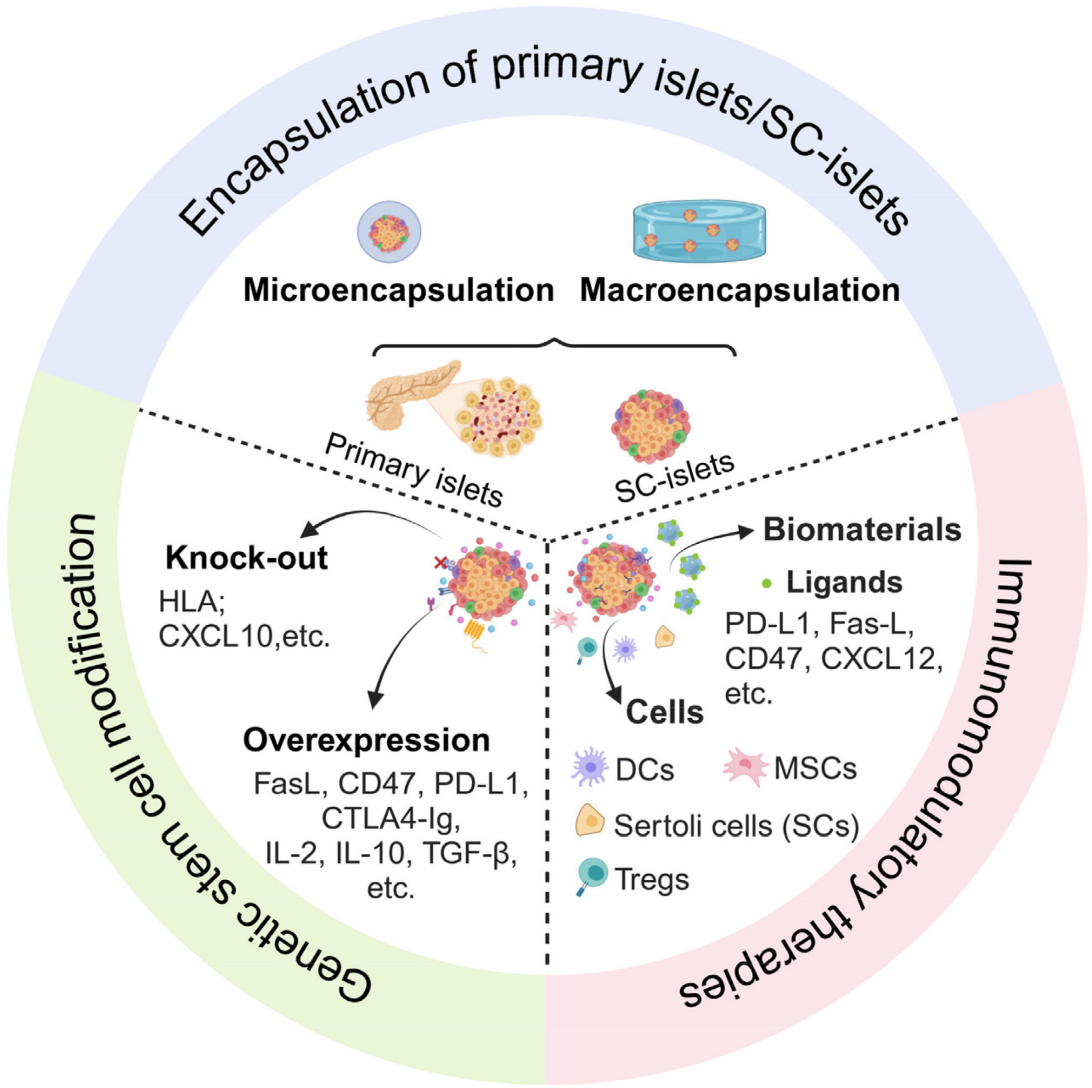
Summary of Strategies to Protect Transplants from Immune Rejection in Treating Diabetes: (1) Encapsulation of primary islets/SC‐islets; (2) Genetic stem cell modification; (3) Immunomodulatory therapies (Created with BioRender.com).

The presence of autoreactive T and B cells poses a significant challenge to the efficacy and function of transplants [49]. Many strategies exist to protect transplants from immune rejection [50]. In addition to traditional immunomodulatory treatments, alternative strategies such as encapsulation devices, genetic stem cell modifications (universal or hypoimmunogenic stem cells) [51], and multiple innovative immunomodulatory strategies [52] are being investigated to prevent immune rejection in islet or SC‐islet transplantation (Figure 1).

### 3.1 Encapsulation of Primary Islets/SC‐Islets

Immune isolation strategies, also named cell encapsulation, use a physical barrier to protect allogeneic primary islets or SC‐islets from attacks by the host immune system [53, 54]. This semipermeable membrane facilitates the exchange of nutrients and oxygen while preventing the infiltration of inflammatory cells and large molecules, such as antibodies and complement proteins. Over the past few decades, polymers have been developed for encapsulating primary islets or SC‐islets and tested in various preclinical animal models [55, 56, 57, 58, 59]. Further optimized polymers are developed to mitigate the foreign body reaction (FBR). In the following section, we review the perspectives and ongoing challenges associated with biomaterial applications. There are primarily two types of encapsulation devices—micro‐ and macro‐encapsulation devices—with recent progress summarized in Tables 1 and 2.

**TABLE 1:** Different Materials for Micro‐Encapsulation of Primary Islets/SC‐Islets.

Biomaterials		Grafts	Results	Ref
Natural	Chitosan	Primary Wistar rat islets	T‐cell lineages and the monocyte/macrophages immunostaining tested negative (4‐week)	[67]
	Chitosan	Primary porcine islets	Pericapsular fibrosis was significantly less	[68]
	Alginate	Primary human islets	Anti‐MHC class I‐II and GAD65 antibodies tested negative (3‐year)	[69]
	Alginate	SC‐β cells	Co‐encapsulated with CXCL12 evaded the pericapsular fibrotic response	[70]
	Modified alginate	Primary rat islets; SC‐β cells	Hypo‐immunoreactive; Fibrosis‐mitigating	[71]
	Agarose	Primary nonobese diabetic (NOD) islets	No mononuclear cellular infiltration	[72]
Synthetic	Polyethylene glycol (PEG)	Primary rat islets	No immune cell infiltration into the hydrogel	[73]
	Triazole‐zwitterionic (TR‐ZW)	Primary rat islets	Lower FBR	[74]
	Polyvinyl alcohol (PVA)	Primary Wistar rat islets	PVA could prevent cell penetration and escape	[75]

**TABLE 2:** Different Materials for Macro‐Encapsulated of Primary Islets/SC‐islets.

Bio‐materials	Grafts	Results	Ref
Autologous collagen‐covered device	Primary porcine islets	No complications and porcine endogenous retrovirus infection were detected	[80]
TheraCyte device	Neonatal pancreatic tissue	Devices effectively protected against β‐cell‐specific immune attacks	[81]
βAir device	Primary human pancreatic islets	Device was safe and successfully prevented immunization and rejection of transplants	[82]
Nanofibrous device + alginate hydrogel	Primary rat islets SC‐β cells	FBR, fibrotic tissue was decreased	[71]
Nanofibrous skin + hydrogel core	SC‐β cells	Little xenogeneic immune responses	[77]
SONIC device	Primary rat islets	Maintain high viability	[83]
VC‐01	hESC‐derived pancreatic progenitor cells	Device had no evidence of allogeneic or autoimmune rejection or sensitization	[84]
VC‐02	hESC‐derived pancreatic progenitor cells	Safe and well‐tolerated; Observed no HLA sensitization	[37]
VX‐264	hiPSC‐derived β cells	Ongoing clinical trial	[85]
iBEDv3S devices	Primary rat islets	Enhance cellular O_2_ supply; fewer T cells and macrophages	[86]
TRAFFIC device	Primary rat islets; Primary human islets	Minimal cell attachment and fibrosis	[87]
VX‐880	Allogeneic SC‐β cells	Most of them were mild to moderate adverse events	[88]
Zwitterionic polyurethanes (ZPU device)	Primary rat islets	Lower FBR or cellular overgrowth	[78]
Cell Pouch device	SC‐β cells	No scar tissue formation was found	[47]
SHEATH system	Primary rat islets primary human islets	Improve local O_2_ environment and create a vascularized subcutaneous cavity	[89]

#### 3.1.1 Micro‐Encapsulation

Micro‐encapsulation involves using biomaterials with a thin layer that allows the exchange of nutrients, oxygen, and metabolites. The materials commonly used for islet microencapsulation include natural polymers such as chitosan hydrogel, alginate, agarose, and others (see Table 1). Currently, alginate is considered an ideal material for microencapsulation [60]. This method involves encapsulating a small number of pancreatic islets (typically fewer than three) and has been successfully validated in mouse [61], NHP [62], and human models [63]. However, challenges persist with post‐implantation. Variability in batch‐to‐batch production and changes in biomaterial composition can affect the gelling process, mechanical behavior, and encapsulation stability, potentially triggering different foreign body reaction (FBR) responses.

Synthetic polymers, such as polyethylene glycol (PEG), are not affected by the same issues encountered with natural polymers (see Table 1). Additionally, synthetic hydrogels generally exhibit excellent mechanical strength, chemical stability, and greater adjustability [64]. As a result, researchers are increasingly interested in using synthetic polymer‐based hydrogels to reduce immune rejection. PEG‐based hydrogels are particularly promising for islet encapsulation due to their lower fibrotic responses [65]. However, their susceptibility to degradation limits their widespread application in vivo [66]. Although other synthetic hydrogel materials, such as polyvinyl alcohol (PVA), have been explored, factors like the conditions during the gelation process (e.g., ultraviolet radiation) can adversely affect the viability of encapsulated transplants, also limiting their in vivo use. In addition, due to the poor mechanical properties of hydrogels, a large number of micro‐encapsulation devices need to be implanted to achieve a sufficient cell quantity, and it is difficult to remove all of them, posing certain safety risks, too.

#### 3.1.2 Macro‐Encapsulation

Macro‐encapsulation devices are generally easier to manufacture, retrieve and commercialize compared with microencapsulation devices [76]. It has received widespread attention in the field of cell therapy due to its advantages, such as high cell loading capacity and the ability to be completely retrieved. Examples include the TheraCyte, βAir, and VC‐02 devices and so on (see Table 2). These commercial devices are designed to prevent the entry of immune cells, thereby achieving a certain degree of immune tolerance without the need for immunosuppression. To address the core challenges in the field of cell therapy, particularly immune rejection, we have conducted extensive research over the past decade employing advanced engineering technologies. For example, by using a highly porous and durable nanofibrous skin made by electrospinning a biocompatible medical‐grade thermoplastic silicone‐polycarbonate‐urethane (TSPU) and an alginate hydrogel core, we developed an implantable nanofiber‐integrated cell encapsulation (NICE) device that offers enhanced biocompatibility, safety, and scalability for large‐scale production, ensuring the safe delivery and protection of xenogeneic stem cell‐derived islets [77]. To further improve the biocompatibility of the encapsulation device in large animals, our team previously reported a zwitterionic polyurethanes (ZPU) nanoporous encapsulation device using electrospinning technique. This device demonstrated excellent anti‐foreign body reaction capabilities in the peritoneal cavities of experimental dogs and pigs [78]. Several macro‐encapsulation devices, including PEC‐Encap (NCT02239354) and PEC‐Direct (NCT03162926, NCT03163511), have been developed for clinical trials using prevascularization polytetrafluoroethylene/polyester membranes. However, while PEC‐Direct encapsulation device enables direct vascularization of the implanted cells, this approach cannot enable immune protection, thereby necessitating the chronic use of immunosuppressive agents. Above all, macro‐encapsulation has several drawbacks: (1) Prone to aggregation: Cells within devices can aggregate into large clusters, which may lead to core necrosis of the cluster; (2) Limited mass transfer: There is restricted exchange of nutrients and gas, which can adversely affect the viability of the islets [79]; (3) Weak structural integrity: The packaging structure may be weak and prone to rupture; (4) Small surface‐to‐volume ratio: This can limit the efficiency of nutrient and gas exchange; (5) Poor and delayed vascular regeneration in transplants is the key issue in pancreatic islet transplantation. Consequently, both academic laboratories and commercial companies are focused on optimizing the balance between preventing immune rejection and ensuring adequate angiogenesis and oxygenation to maximize the quality and function of transplanted islets in future clinical applications.

Encapsulation technology offers immune protection for islets by creating a barrier that prevents immune cell attack, while still allowing the islets to secrete insulin and absorb necessary nutrients. Micro‐encapsulation has shown promising results in animal models, whereas macro‐encapsulation has achieved significant breakthroughs in clinical trials. A key challenge in advancing encapsulation technology for clinical use is to improve the biocompatibility of the biomaterials and minimizing the fibrosis induced by membranes. With advancements in materials science and nanotechnology, encapsulation technology poses the potential to provide sustained immune protection for islet transplantation and reduce the reliance on immunosuppressive drugs in the future.

### 3.2 Genetic Stem Cell Modification

Since the use of genetic manipulation has become widespread, strategic gene editing of human pluripotent stem cells (hPSCs) is increasingly employed to generate stem cell‐derived islets that minimize immune responses. Graft rejection primarily arises from T cell recognition of donor major human leukocyte antigens (HLAs), also known as the major histocompatibility complex (MHC) in humans. Classical HLAs include class I genes (HLA‐A, ‐B, and ‐C), which are expressed on most nucleated cells, and class II genes (HLA‐DR, ‐DP, and ‐DQ), which are expressed on T cells, B cells, and antigen‐presenting cells [90, 91]. In addition, nonclassical HLA class I molecules are composed of HLA‐E, ‐F and ‐G [92]. Among these, HLA‐A, ‐B, and ‐C are the most studied factors related to allorejection [93]. HLA‐G is well known in fetal–maternal tolerance [94] and transplantation [95] to maintain immune homeostasis. It is crucial to generate universal hPSCs that do not express HLA molecules. Therefore, we will discuss strategies for preparing universal or hypoimmunogenic hPSCs.

#### 3.2.1 Knock‐Out of Immunomodulatory Molecules

In vitro engineering techniques, such as the depletion or downregulation of specific molecules, are being explored to mitigate potential immune responses in iPSC‐derived graft transplantation. Knocking out or knocking down β‐2‐microglobulin (β2M) can limit the formation of heterodimers between β2M and HLA class I proteins, thereby preventing the expression of HLA class I proteins and reducing the immune response from cytotoxic CD8+ T cells [96, 97]. Additionally, multiple clustered regularly interspaced short palindromic repeats (CRISPR)/CRISPR‐associated protein 9 (Cas9) techniques can be used to simultaneously knock out HLA‐A, ‐B, and ‐C, achieving complete HLA class I depletion [98]. However, knocking out β2M or depleting HLAs can increase the sensitivity of hPSCs to natural killer (NK) cells and macrophages due to the loss of self‐recognition [99]. In addition, single‐cell RNA sequencing and CRISPR‐based genome‐wide screening has more broad applications in SC‐islets transplantation. Sintov et al. [100] found knocking out chemokine ligand 10 (CXCL10) reduced the immunogenicity of SC‐islets through manipulating the activity of the Janus kinase/signal transducer and activator of transcription (JAK/STAT) pathway. Therefore, it is crucial to develop multiple strategies that enable hPSCs to avoid both adaptive and innate immune reactions.

#### 3.2.2 Overexpression of Immune Checkpoint Inhibitors

Universal hPSCs engineered to express specific genes can evade detection by T cells, NK cells, and macrophages. For example, CD95‐CD95L (also known as Fas–Fas ligand) pathway is crucial for regulating T‐cell responses, an important strategy for local immune evasion of fetal trophoblast cells [101]. Engineering of FasL on the surface of primary mouse islets has enabled these islets to survive without immunosuppression by reducing the proliferation of alloreactive T cells and macrophages [102]. Another example involves CD47/SIRPα pathway, known as the “don't eat me” signal, which is highly expressed in tumor cells and interacts with receptors to prevent phagocytosis [103]. Deuse et al. designed hPSCs in which β2M and CIITA were depleted, and CD47 was subsequently knocked in using CRISPR/Cas9 technology (B2M^−/−^/CIITA^−/−^/CD47^+^) [104]. This modification allowed cardiomyocytes and endothelial cells to escape immune responses, inhibit NK cell activation, and survive effectively in humanized mice without immunosuppression. Additionally, the overexpression of programmed death‐ligand 1 (PD‐L1) in hPSCs protects SC‐islets from immune rejection by restricting T cell activation after transplantation in mouse model [105].

However, in contrast to previous studies, Gerace et al. reported that targeting HLAs and PD‐L1 was insufficient to protect transplanted SC‐islets from allogeneic immune responses [106]. To address this, they engineered SC‐islets to secrete several immunomodulatory factors: modified IL‐2 to facilitate Treg expansion, and IL‐10 and TGF‐β to promote tolerogenic local microenvironment, thus enhancing Treg immunosuppressive functions. These modified SC‐islets demonstrated effective survival in NOD mouse model. Additionally, various eight immunomodulatory factors, including PD‐L1, CD200, CD47, H2‐M3, FasL, Serpinb9, CCL21, and MFGE8, have been induced to express in mouse embryonic stem cells (mESCs) and human pluripotent stem cells (human HLA‐G replaced mouse H2‐M3) to create genetically engineering hypoimmunogenic cells or artificial tissues derived from them [107].

Above all, these approaches could potentially generate universal or hypoimmunogenic hPSC sources for future transplantation of hPSC‐derived islets, thereby minimizing reliance on immunosuppressive agents and reducing the need for encapsulation. Nevertheless, the microenvironment of SC‐islet transplantation in vivo is more complex; further investigation into specific tolerogenic mechanisms is essential to ensure the long‐term safety and efficacy of SC‐islet transplantation therapies in clinical practice for T1D.

Although gene editing technologies remain in the preclinical research phase, their potential is substantial. Achieving immune evasion of islet cells in clinical settings would constitute a revolutionary advancement in the field of islet transplantation.

### 3.3 Immunomodulatory Therapies

As we know, long‐term use of immunosuppressants is required to prevent the rejection of primary or SC‐islet grafts. However, these medications can be toxic to the islets and harmful to patients. Today, alternative strategies to eliminate immunosuppression are being explored by using biomaterials‐mediated immunomodulation and immunomodulatory cell co‐transplantation approaches. Despite their benefits in reducing immune reactions, systemic immunomodulation can lead to significant adverse effects [108]. Local immunomodulatory strategies that effectively protect transplanted islet function while minimizing side effects are highly encouraged. Unlike encapsulation, local immunomodulation does not involve a physical barrier between the grafts and the body, aiming to facilitate better and more direct host integration.

#### 3.3.1 Biomaterials‐Mediated Immunomodulation

Recently, strategies employing biomaterials‐engineered ligands have emerged for manipulating islets to enhance immune protection. For instance, the modification of islet surfaces with streptavidin (SA)‐FasL‐functionalized PEG microgels in a T1D mouse model for allogeneic islet transplantation has demonstrated long‐term survival and function by inducing and expanding CD4+/CD25+/Foxp3+ Tregs without chronic immunosuppression [109, 110]. Similarly, the transplantation of allogeneic islets mixed with SA‐PD‐L1‐presenting microgels into various models, including diabetic mice [111], NOD mice [112], and diabetic non‐human primates (NHP) [113], has achieved prolonged survival and function of allogeneic islets. In addition to immunomodulatory agents (such as FasL and PD‐L1), other materials used for islet delivery include biological agents (CTLA4‐Ig), and chemokines (CXCL12, IL‐2, TGFβ1) [114]. These results highlight significant potential for broader clinical application. However, despite advances in immunomodulatory biomaterial modifications, transplanted islets continue to face challenges such as immune rejection, inadequate vascularization, hypoxia, and fibrosis, which compromise their viability and function. The application of these materials to human clinical trials remains a considerable challenge.

#### 3.3.2 Co‐Transplantation With Immunomodulatory Cells

Increasing interest is being directed toward cell co‐transplantation strategies for local immune modulation, including the use of mesenchymal stromal cells (MSCs), Tregs, dendritic cells (DCs), and Sertoli cells (SCs) [115]. These approaches aim to enhance the survival and function of transplanted islets while minimizing the need for systemic immunosuppression.

(1) MSCs.

Co‐transplantation of MSCs has shown potential in promoting long‐term graft survival primarily by reducing inflammation and enhancing immune tolerance [116, 117]. Li et al. found that co‐transplanting MSCs with allogeneic islets decreased Th1/Th2 ratio inhibited DC maturation and suppressed IL‐12 secretion [118]. Furthermore we reported another notable approach involves the co‐transplantation of engineered MSCs (eMSCs) that overexpress PD‐L1 and cytotoxic T lymphocyte antigen 4 immunoglobulin (CTLA4‐Ig) with islets. This strategy has demonstrated promise in improving long‐term graft survival by reducing CD4+ and CD8+ effector T cell (Teff) infiltration and increasing the number of regulatory T cells (Tregs) [119]. Although these studies suggest that MSC co‐transplantation plays a significant role in reducing immune rejection several challenges remain: (1) Further research is needed to establish the long‐term safety of MSCs in clinical practice and (2) Ensuring the stability of MSC function over time remains uncertain.

(2) Tregs.

Tregs play a crucial role in preventing autoimmune rejection [120]. For instance, co‐transplanting Tregs with islets has been shown to significantly prolong islet survival in diabetic mice by protecting the islets from autoimmune destruction [121]. Furthermore, using poly(lactide‐co‐glycolide) (PLG) scaffolds for co‐transplantation of Tregs and islets can extend long‐term graft survival through the action of host‐derived Tregs [122]. Patients who received Tregs alone maintained higher fasting C‐peptide levels compared to an untreated control group [123]. Despite these promising developments, the clinical application of Tregs in conjunction with primary pancreatic islets still requires further exploration. Currently, no standardized method for large‐scale production and long‐term maintenance of the Treg function has been established. If successful, such clinical trials could substantially benefit T1D patients undergoing islet transplantation.

(3) DCs.

The key factor in allograft immune rejection is the activation of DCs, which leads to a T cell‐mediated adaptive immune response [124]. Immune tolerance is primarily induced by immature, tolerogenic DCs (tolDCs) [125]. Therefore, tolDC‐based therapies are emerging as promising strategies for managing allograft rejection. These include approaches such as using host immature DCs, in vivo allopeptide‐primed DCs, drug‐treated mature autologous DCs, and gene‐modified DCs (e.g., IL‐10, CTLA4‐Ig, and GAD65/DCR3) [115]. For example, co‐transplanting DCs pre‐treated with MSCs with pancreatic islets has been shown to reduce immune rejection by inhibiting MHC‐II expression [126]. However, the broader application of nanoparticles is limited by their high cost. Similarly, challenges remain in the generation and maintenance of both DCs and Tregs for clinical use.

(4) *SCs*.

The testis is a natural immune‐privileged site that supports the long‐term survival of allogeneic and xenogeneic transplants. Sertoli cells (SCs) are particularly crucial in establishing an immune‐tolerant environment at the blood‐testis barrier (BTB), where they highly express immune‐modulating factors such as Fas ligand (FasL), TGF‐β, and clusterin [115, 127]. For example, co‐transplanting SCs with primary islets has been shown to significantly enhance islet survival [128, 129, 130, 131]. Additionally, using a subcutaneous collagen‐covered device loaded with both neonatal porcine islets and porcine SCs in Type 1 Diabetes (T1D) patients has successfully avoided immune responses without the need for immunosuppression [128]. Recent research has introduced a method for in vitro reprogramming of fibroblasts into human‐induced Sertoli‐like cells (hiSCs) using a stable transgenic approach with just two transcription factors: NR5A1 and GATA4 [132]. This method provides a source of portable SCs from alternative origins. SCs offer several advantages in future cell therapies, including their terminal differentiation properties, which mitigate concerns about malignant expansion after transplantation and alleviate related ethical issues. Despite their success in human clinical studies for co‐transplantation with pancreatic islets, the long‐term safety of SCs remains uncertain, and there is limited research on the use of SCs derived from other sources.

As previously mentioned, various strategies exist to delay or prevent immune rejection during islet transplantation. A combination of multiple strategies is often employed in research. Recently, Heon‐Seok Park et al. investigated the long‐term efficacy of several encapsulation methods, including naked, alginate, alginate‐chitosan, alginate‐perfluorodecalin, and alginate‐chitosan‐perfluorodecalin (AC‐PFD), for delivery of wild type, or α1,3‐galactosyltransferase knockout (GTKO) porcine islets to achieve immune evasion. They found that GTKO islet transplantation in diabetic mice model using AC‐PFD capsules resulted in the most favorable long‐term outcomes, compared to wild type genetic traits, effectively maintaining normal blood glucose levels for up to 180 days while ameliorating hypoxia and inflammation [133]. Despite the success in creating local immune‐tolerant microenvironments, the clinical adoption of co‐transplantation involving immune regulatory cells and pancreatic islets still requires further development. This is largely due to several challenges: the high demand for immune regulatory cells, variability in cell survival rates and implantation methods, and the need to preserve their functionality after implantation.

Above all, the future of immune protection strategies in islet transplantation is poised to evolve toward a more integrated and precise approach. The combination of various strategies, such as the use of tol‐DCs with gene editing and stem cells with encapsulation technologies, holds significant potential for establishing a more stable immune tolerance environment. With continuous advancements in bioengineering, nanotechnology, and genomics, these strategies are expected to see broader clinical application, providing more sustainable and effective therapeutic options for diabetes patients.

## 4 Conclusion, Challenges, and Perspectives

Blood glucose levels in humans are primarily regulated by insulin secreted by pancreatic β‐cells. For clinical T1D treatment, exogenous insulin administration cannot fully replicate the regulatory function of endogenous pancreatic β‐cells [134], highlighting the urgent need for innovative therapeutic strategies aimed at curing diabetes. Currently, approved islet cell replacement therapies include both pancreas and islet transplantation. Pancreas transplantation procedure is both complex and technically demanding. In contrast, islet transplantation is less invasive, involves simpler clinical procedures, and generally has a higher safety profile. Human islet transplantation has substantially improved glycemic management and restored insulin independence in patients with T1D. However, several substantial challenges remain, including: (1) the adverse effects of long‐term immunosuppression. Chronic immunosuppression increases the susceptibility to infections due to weakened body's immune system, reducing its ability to combat pathogens. (2) the risk of immune rejection of the allogeneic graft. Allogeneic cells are recognized by the recipient's immune system, triggering an immune response that leads to the activation of immune cells and subsequent rejection of the transplanted cells. This immune‐mediated attack ultimately results in graft failure. Both primary islet transplantation and surrogate organ/cell therapies necessitate strategies to minimize or eliminate immunosuppression. This review summarizes progress in three main approaches: (1) encapsulation technologies to prevent immune rejection, (2) gene‐editing strategies of stem cells, and (3) immunomodulation strategies. Integrating multiple strategies, such as delivering genetically edited stem cells with open devices to enhance immune tolerance, safety, and function, is critical in current and future preclinical and clinical endeavors.

Despite promising results in various animal models, the translation from bench work to clinical applications requires rigorous assessment of functionality and safety. Consequently, there is an urgent need for more clinical data to drive the innovation of immune protection strategies in the transplantation. Certainly, the existing clinical data also provide valuable insights: (1) For widespread clinical application in the future, a large number of islets will be required for each patient. Recent work by Wang et al. in Cell reports a functional cure of a T1D patient after CiPSC‐islet transplantation with 1 488 283 IEQ (19 843 IEQ/Kg, 1 IEQ contains approximately 1348.5 ± 187.6 CiPSC‐islet cells) [44]. This highlights the urgent need for efficient, high‐purity differentiation of SC‐β cells and scalable, automated production to meet GMP standards. To overcome this challenge, selecting appropriate stem cell lines and optimizing differentiation efficiency through the modulation of growth factors and small molecules are critical steps in the process. In high‐efficient differentiation protocols, it is necessary to incorporate appropriate cell purification methods. By integrating the complete workflow of differentiation, purification, enrichment and maturation of SC‐β cells, both the purity and safety of the final cell product can be improved significantly. (2) To address long‐term safety and immune rejection, effective cell encapsulation devices can be developed to establish immune barriers while permitting the exchange of small molecules such as nutrients, glucose, oxygen, and insulin. The development of functional large‐scale encapsulation devices for delivering SC‐islets subcutaneously to T1D patients holds significant promise. The pore size of the devices must be carefully controlled to prevent the escape of SC‐islets into the host, ensuring their safety. (3) Beyond islet transplantation, gene‐editing strategies to enable stem cells derivatives to evade immune recognition offer substantial potential, though it remains a puzzle in identifying functional immunosuppressive targets and validating them in vivo for clinical application. (4) Currently, islet transplantation is predominantly performed via the hepatic portal vein. However, recent studies have also explored alternative transplantation sites, including adipose tissue [135, 136]. Notably, clinical trial on SC‐islets transplantation under the abdominal anterior rectus sheath indicates promising outcomes in this transplantation site [44]. Nevertheless, alternative extra‐portal sites for islet transplantation require further validation through additional clinical studies. The integrated use of various strategies, including gene editing, immune modulation, cell encapsulation, immune tolerance induction, and co‐transplantation, can effectively sustain local immune tolerance at the transplantation site, thereby offering feasible solutions to improve the success rate of islet transplantation.

To address these critical issues, we propose a range of potential strategies designed to enhance the efficacy and applicability of this therapeutic approach. One critical strategy involves improving the immune protection of transplanted islets. This may be achieved through the development of advanced encapsulation devices that safeguard islets from immune‐mediated rejection, in conjunction with the use of immunomodulatory therapies to further minimize the likelihood of graft rejection. Another promising direction is the enhancement of graft survival through the exploration of optimized biomaterials and devices that support islet function and stability following transplantation. Additionally, optimizing vascularization at the transplantation site is essential to ensuring an adequate supply of nutrients and oxygen to the graft, thereby promoting its long‐term viability. Furthermore, bioengineered SC‐islets with inert immune responses represent potential solutions to both the ongoing challenge of donor organ scarcity and immune rejection.

In summary, the strategies discussed above aim to minimize immune recognition and rejection of transplanted islet cells. By integrating these approaches, we aim to identify pivotal breakthroughs that will accelerate the clinical translation of islet transplantation, ultimately making this therapy more accessible, effective, and scalable for patients with diabetes. Furthermore, these strategies hold potential beyond diabetes, as they could be applied to a wide range of other cell‐based therapies, thereby benefiting a broader patient population with diverse medical complications.

## Conflicts of Interest

The authors declare no conflicts of interest.
